# Trends of stillbirth among reproductive-age women in Ethiopia based on Ethiopian demographic and health surveys: a multivariate decomposition analysis

**DOI:** 10.1186/s12884-020-02880-5

**Published:** 2020-03-30

**Authors:** Getayeneh Antehunegn Tesema, Lemma Derseh Gezie, Solomon Gedlu Nigatu

**Affiliations:** Department of Epidemiology and Biostatistics, Institute of Public Health, College of Medicine and Health Sciences, University of Gondar, Gondar, Ethiopia

**Keywords:** Ethiopia, Multivariate decomposition analysis, Trends, Stillbirth

## Abstract

**Background:**

Despite the effort to reduce stillbirth, Ethiopia remains one of the countries with the highest rate in the world. Therefore, this study aimed to analyze the trends of stillbirth among births from reproductive age women over time based on Ethiopian Demographic and Health Surveys (EDHSs).

**Methods:**

Secondary data analysis was conducted based on the Ethiopian Demographic Health Surveys (EDHSs) conducted in 2005, 2011 and 2016. A total weighted sample of 12,037, 10,588, and 11,375 in 2005, 2011 and 2016 respectively were included for analysis. Trend and Logistic based decomposition analysis technique was used for analyzing the trends of stillbirth over time and factors contributing to the change in stillbirth rate. STATA 14 was employed for data management and analyses. All analyses presented in this paper were weighted for the sampling probabilities and non-response. Complex sampling procedures were also considered during testing of statistical significance.

**Results:**

Among women of reproductive age, the stillbirth rate declined from 13.3/1000 births in 2005 to 9.2 per 1000 births in 2016 with the annual rate of reduction of 3.1%. The study found that the stillbirth rate has been declined over time concerning the place of residence, region, antenatal care, education and place of delivery. The decomposition analysis indicated that about 82.3% of the overall change stillbirth rate was due to the difference in women’s composition. Particularly, an increase in women’s urban place of residence, health facility delivery, and cesarean delivery were significant predictors for the decline in stillbirth rate over the surveys.

**Conclusions:**

The stillbirth rate has been declined over time. More than 3/4th of the decrease in stillbirth rate was due to the difference in characteristics of women over the surveys. The increase in women’s urban place of residence, an increase in cesarean delivery and health facility delivery significantly contributed to the decrease in stillbirth rate over time. Public health interventions targeting rural resident women, strengthening emergency obstetric services and health facility delivery would help to maintain the decreasing trend of stillbirth rate in Ethiopia.

## Background

For international comparisons, the World Health Organization (WHO) defines stillbirth as a baby born with no sign of life at or after 28 weeks of gestation or birth weight of 1000 g or more or body length of 35 cm or more [1]. Globally in 2015, an estimated 2.6 million third trimester stillbirths, of these more than 98% occurred in low and middle-income countries and over three-quarters of these occurring in Sub-Saharan Africa (SSA) and South Asia [2]. India, Pakistan, Nigeria, China, Bangladesh, the Democratic Republic of the Congo, Ethiopia, Indonesia, Tanzania, and Afghanistan are the only 10 countries that carry the burden of over 65% of total stillbirths in the world and Ethiopia has been ranked in the seventh position [2, 3].

Stillbirth is twice as common as neonatal mortality [4] and It has reduced more slowly than maternal mortality or mortality in children younger than 5 years, which remain invisible in global policies [2] with an annual Average Rate of Reduction (ARR) of 2.0% in comparison to ARR of 3.0% for maternal death or 3.1% for neonatal death [5]. It is the commonest adverse pregnancy outcome that is less accounted for and gets relatively lower attention at both policy and implementation levels [6]. For instance, stillbirths are not accounted for in the Global Burden of Disease and the Millennium Development Goals (MDGs). Even the recently Established Sustainable Development Goals (SDGs) and targets that have been declared in 2015 did not have any focused commitment to reduce stillbirth [7].

Though Ethiopia has free obstetric services (including basic and emergency obstetric services) and prenatal care, the local health system lacks adequate qualified staff, functional equipment, and ambulance for emergency referral especially for rural residents and communities poor attitude towards maternal health service utilization makes stillbirth to not reduce as expected [8]. According to the systematic review of studies done on 16 hospital-based and few community-based studies in Ethiopia published between 1977 and 2012 the rate of stillbirth was declined from 34 to 28 per 1000 live births [9].

Stillbirth remains a public health problem, especially in developing countries where rates are 10-fold higher than in developed countries. The United Nations Every New Born Action Plan (ENAP) has set a target of 12 stillbirths per 1000 births by 2030 and 10 stillbirths per 1000 births by 2035 [10, 11]. Ninety-two mainly high-income countries have already met this target, although with marked disparities. At least 67 countries, particularly in Africa and conflict-affected areas will have to double current progress [12, 13]. It is the main indicator of the quality of the health services that are provided during pregnancy and childbirth [14].

The long-lasting impact of stillbirth remains a large burden for parents, families, policymakers and public health practitioners [12]. Evidence has shown that stillbirth is associated with physical, social and psychological morbidity, and remains a significant source of cost for the affected family and community [15]. Moreover, the complex socio-cultural consequences including grief, stigma, blame, marginalization, and anxiety which often lasts a long period are believed to bear considerable emotional and mental health effects [16]. Despite the huge burden of stillbirth on families and global health, progress made in low-middle-income countries to reduce stillbirth is considerably slower than the decline in child mortality [6]. Experiencing stillbirth had a huge impact on affected families; they suffer from grief and anxiety which often lasts a long period.

Despite achieving remarkable results in many health indicators including reducing maternal and child mortality over the last decade, Ethiopia remains one of the countries with the highest rate in the world despite relatively improved access to maternal health service over time. Although the magnitude of stillbirth in Ethiopia was among the highest in Sub Saharan Africa, stillbirth remains invisible and neglected in health policies and programs has reduced more slowly than maternal mortality and neonatal mortality [9]. Neither the Health Management Information System (HMIS) nor Ethiopian Demographic Health Surveys (EDHS) reports the magnitude and trends of stillbirth over time [17–19].

Different studies done on determinants of stillbirth showed that rural residence, parity, educational status, mode of delivery, ANC utilization, and place of delivery, maternal nutritional status, and maternal obstetric factors were the significant predictors of experiencing stillbirth [20–23]. Previous studies utilized only one point survey data [9, 24–26]; it is difficult to observe the trend and to identify factors that have been consistent in influencing stillbirth over time. Studying the change in stillbirth using multivariate decomposition analysis to identify determinant factors associated with the change in stillbirth over time has become relevant for targeting interventions to work on factors contributing to the decrease in stillbirth and could critically inform policies and programs aimed at reducing stillbirth in Ethiopia. Therefore, this study addressed such gaps by investigating the magnitude and trends as well as determining the factors contributing to the change in the Stillbirth rate over time using a Multivariate Decomposition Analysis based on 2005, 2011 and 2016 Ethiopian Demographic and Health Surveys (EDHSs).

## Method and materials

### Data

This study was a secondary data analysis based on 2005, 2011 and 2016 Ethiopian Demographic and Health Surveys (EDHS). The EDHS used a stratified two-stage cluster sampling technique selected in two stages using the 1994 Population and Housing Census (PHC) frame for EDHS 2005, and 2007 the Population and Housing Census (PHC) frame for EDHS 2011 and 2016 as a sampling frame. Stratification was achieved by separating each region into urban and rural areas. In total, 21 sampling strata have been created because the Addis Ababa region is entirely urban. In the first stage, 540 Enumeration Areas (EAs) (145 in the urban area) for EDHS 2005, 624 EAs (187 in the urban area) for EDHS 2011 and 645 EAs (202 in the urban area) for EDHS 2016 were selected with probability proportional to the enumeration areas size and with independent selection in each sampling stratum. At the second stage, because the time has passed since the PHC, a complete household listing operation was carried out in all selected EAs before the start of fieldwork and on average 28 households were systematically selected. The detailed sampling procedure was presented in the full EDHS report [17–19]. The source of the population was all births from reproductive-age women in Ethiopia whereas all births from reproductive-age women in the selected enumeration areas were the study population.

### Study variables

#### Outcome variables

The EDHS asked women to report any pregnancy loss that occurred in the 5 years preceding the survey. For each pregnancy that did not end in a live birth, the duration of the pregnancy was recorded. Pregnancy losses occurring after seven completed months of gestation are defined as stillbirths. The response variable of this study is the occurrence of stillbirth among mothers of childbearing age.

The response variable for the i^th^ mother is represented by a random variable Y_i_ with two possible values coded as 1 and 0. So, the response variable of the i^th^ mother Y_i_ was measured as a dichotomous variable with possible values Y_i_ = 1, if _ith_ mother had experienced stillbirth and Y_i_ = 0 if mother had a live birth.

#### Independent variables

Socio-demographic and economic variables (residence, region, maternal age, marital status, religion, maternal education, paternal education, wealth index, maternal occupation, maternal working Status), Pregnancy and pregnancy-related factors (Mother’s height, BMI, ANC visit, Parity, Preceding birth interval, contraceptive use, Place of delivery, Birth order, Mode of delivery, wanted pregnancy, Maternal anemia), Behavioral factors (Smoking, media exposure) were included for this study.

### Data collection procedure

The study was conducted based on EDHS data by accessing from the DHS program official database www.measuredhs.com after permission was granted through an online request by explaining the objective of our study. The raw data was collected from all parts of the country on childbearing aged women using a structured and pre-tested questionnaire. We used the Birth Record (BR file) data set and extracted the outcome and independent variables.

### Data management and analysis

The data were weighted using sampling weight, primary sampling unit, and strata before any statistical analysis to restore the representativeness of the survey and to tell the STATA to take in to account the sampling design when calculating standard errors to get reliable statistical estimates. Cross tabulations and summary statistics were conducted to describe the study population. Descriptive and summary statistics were conducted using STATA version 14 software.

Data from EDHS 2005, 2011 and 2016 were appended together after extracting important variables for trend and decomposition analysis.

### Trend and decomposition analysis

The trend was assessed using descriptive analyses stratified by selected respondent characteristics and was assessed separately for the periods 2005–2011, 2011–2016, and 2005–2016.

A multivariate decomposition analysis of the change in stillbirth rate was employed to answer the major factors contributing to the difference in the percentage of stillbirth over the study period. This method is used for several purposes in economics, demography, medicine and other specialties. The present analysis focused on how the stillbirth rate responds to differences in women’s characteristics and how these factors shape the differences across surveys conducted at different times. The analysis was a regression analysis of the difference in the percentage of stillbirth rates between EDHS 2005 and 2016. The purpose of multivariate decomposition analysis was to identify the source of difference in the percentage of stillbirth in the last 10 years. Both the difference in composition (Endowment) of population and difference in the effect of the characteristics (Coefficient) between the surveys is important to know the factors contributing to the decrease in stillbirth rate over time. The multivariate decomposition analysis for nonlinear response models utilizes the output from a logistic regression model since it is “a binary outcome” to parcel out the observed difference in stillbirth rate between the surveys into components. The difference in the rate of stillbirth between the surveys can be attributed to the compositional difference in population (difference in characteristics or endowment) and the difference in the effect of explanatory variables (difference in coefficient) between the surveys.

Logit based decomposition analysis technique was used for the analysis of factors contributing to the change in stillbirth over time to identify factors contributing to the change in stillbirth rate in the last 10 years.

The changes of stillbirth over time can be attributed to the compositional difference between surveys and differences in the effects of the selected explanatory. Hence, the observed difference in stillbirth between surveys is additively decomposed into a characteristics (or endowments) component and a coefficient (or effects of characteristics) component.

For logistic regression, the Logit or log-odd of stillbirth is taken as:

$$ {\displaystyle \begin{array}{l}\mathrm{Logit}\ \left(\mathrm{A}\right)-\mathrm{Logit}\ \left(\mathrm{B}\right)=\mathrm{F}\ \left(\mathrm{XA}\upbeta \mathrm{A}\right)-\mathrm{F}\ \left(\mathrm{XB}\upbeta \mathrm{B}\right)\\ {}=\frac{\left[\mathrm{F}\ \left(\mathrm{XA}\upbeta \mathrm{A}\right)-\mathrm{F}\ \left(\mathrm{XB}\upbeta \mathrm{A}\right)\right]+}{\mathrm{E}}\frac{\Big[\mathrm{F}\ \left(\mathrm{XB}\upbeta \mathrm{A}\right)-\mathrm{F}\ \left(\mathrm{XB}\upbeta \mathrm{B}\right]}{\mathrm{C}}\end{array}} $$

The E component refers to the part of the differential owing to differences in endowments or characteristics. The C component refers to that part of the differential attributable to differences in coefficients or effects.

The equation can be presented as:

$$ \mathrm{Logit}\ \left(\mathrm{A}\right)-\mathrm{Logit}\ \left(\mathrm{B}\right)=\left[\beta 0\mathrm{A}-\beta 0\mathrm{B}\right]+\Sigma \mathrm{XijB}\ast \left[\beta \mathrm{ijA}-\beta \mathrm{ijB}\right]\kern0.5em \Big]+\Sigma \beta \mathrm{ijB}\ast \left[\mathrm{XijA}-\mathrm{XijB}\right] $$

- *Xij*B is the proportion of the jth category of the ith determinant in the DHS 2005,

- *Xij*A is the proportion of the jth category of the ith determinant in DHS 2016,

- *ΒijB* is the coefficient of the jth category of the ith determinant in DHS 2005,

- *ΒijA* is the coefficient of the jth category of the ith determinant in DHS 2016,

- *Β0B* is the intercept in the regression equation fitted to DHS 2005, and.

- *Β0A* is the intercept in the regression equation fitted to DHS 2016.

The recently developed multivariate decomposition for the non-linear model was used for the decomposition analysis of stillbirth using mvdcmp STATA command [27].

## Results

### Characteristics of the study population

About half of the respondents in all three surveys were age 20–29 years. Based on the place of residence, there was a slight increment of urban residents from 7.3% in 2005 to 10.8% in 2016. According to maternal educational status, in survey 2005 more than three quarters (79.3%) of women were not educated, while it was decreased to 69.9 and 66.9% in EDHS 2011 and 2016 respectively (Table 1). Besides, the proportion of women with primary education rose from 16.6 in 2005 to 26.5% in 2011 but slightly decline to 26% in 2016. However, only a small proportion of women had 4 and above ANC visit during pregnancy in 2005 (12.2%), the percentage rose from 12.2 to 58.3% in 2016 and the proportion of women’s with no ANC visit significantly declined from 72% in 2005 to 22.9% in 2016 (Table 1).

**Table 1:** Percentage distribution of characteristics of respondents in 2005, 2011 and 2016 Ethiopian Demographic and Health Surveys

Characteristics		Weighted frequency (%) 2005 *N* = 12,037	Weighted frequency (%) 2011 *N* = 10,588	Weighted frequency (%)2016 *N* = 11,375
Women age	< 20 years	576 (4.7)	361 (3.4)	374 (3.3)
	20–29 years	5825 (48.5)	5429 (51.2)	5599 (49.2)
	30–39 years	4344 (36.2)	3823 (36.1)	4382 (38.5)
	40-49 years	1292 (10.7)	985 (9.3)	1020 (9.0)
Residence	Rural	11,154 (92.7)	9182 (86.7)	10,146 (89.2)
	Urban	883 (7.3)	1406 (13.3)	1229 (10.8)
Region	Tigray	775 (6.4)	678 (6.4)	709 (6.2)
	Afar	122 (1.0)	114 (1.1)	119 (1.1)
	Amhara	2826 (23.5)	2537 (24.0)	2121 (18.7)
	Oromia	4666 (38.8)	4291 (40.1)	4997 (43.9)
	Somalia	522 (4.4)	296 (2.8)	554 (4.9)
	Benishangul-Gumuz	115 (0.9)	131 (1.2)	133 (1.2)
	SNNPRs	2742 (22.8)	2248 (21.2)	2402 (21.1)
	Gambela	34 (0.3)	38 (0.4)	29 (0.3)
	Harari	23 (0.2)	27 (0.2)	27 (0.2)
	Addis Ababa	170 (1.4)	192 (1.8)	234 (2.0)
	Dire Dawa	41 (0.3)	35 (0.3)	49 (0.4)
Religion	Orthodox	5094 (42.3)	4232 (40.0)	3844 (33.8)
	Muslim	4123 (38.3)	3563 (33.6)	4696 (41.3)
	Others^a^	2820 (23.4)	2792 (26.4)	2835 (24.9)
Women education	No education	9541 (79.3)	7404 (69.9)	7606 (66.9)
	Primary education	1993 (16.6)	2801 (26.5)	2961 (26.0)
	Secondary and higher education	503 (4.1)	383 (3.6)	7.1 (808)
Wealth index	Poorest	2646 (21.9)	2420 (22.9)	2483 (24.1)
	Poorer	2497 (20.8)	2382 (22.5)	2623 (23.0)
	Middle	2607 (21.8)	2151 (20.3)	2318 (20.4)
	Richer	2468 (20.4)	2018 (19.1)	2084 (18.3)
	Richest	1819 (15.1)	1616 (15.2)	1613 (14.2)
Maternal occupation	No occupation	8496 (70.6)	5727 (45.9)	6352 (55.8)
	Had occupation	3541 (29.4)	4861 (54.1)	5023 (44.2)
Body Mass Index of women	Thin	7436 (61.7)	2249 (21.2)	2483 (21.8)
	Normal	4401 (36.6)	7668 (72.4)	8164 (71.8)
	Obesity	200 (1.7)	671 (6.4)	728 (6.4)
Ever use contraceptive	Yes	2578 (21.4)	4745 (44.8)	5238 (46.0)
	No	7858 (78.6)	5843 (55.2)	6137 (54.0)
Maternal anemia	Not anemic	4235 (72.3)	8728 (82.4)	7590 (66.7)
	Anemic	1620 (27.7)	1860 (17.6)	3785 (33.3)
Number of ANC visit	No visit	4920 (72.0)	3391 (57.7)	2602 (22.9)
	1–3 visit	1091 (15.8)	1313 (22.3)	2145 (18.9)
	4 and above visit	834 (12.2)	1175 (20.0)	6628 (58.3)
Mode of delivery	caesarean section	103 (1.6)	136 (1.5)	196 (1.9)
	Vaginal delivery	10, 380 (98.4)	9333 (98.5)	9943 (98.1)
Place of delivery	Home	9873 (94.2)	8539 (90.2)	7468 (65.7)
	Health facility	611 (5.8)	930 (9.8)	3907 (34.3)
Maternal height	Less than 150 cm	755 (12.9)	1302 (12.3)	1228 (10.8)
	≥150 cm	5100 (87.1)	9286 (87.7)	10,147 (89.2)
Preceding birth interval	< 24 month	2315 (23.4)	1876 (22.2)	2145 (18.9)
	≥ 24 month	7597 (76.6)	6582 (77.8)	9239 (81.1)
Parity	Only one birth	1197 (9.9)	1115 (10.5)	1419 (12.5)
	2–4 birth	5339 (44.4)	4997 (47.2)	5022 (44.1)
	≥5	5499 (45.7)	4476 (42.3)	4934 (43.4)
Birth order	1–3	5812 (48.3)	5415 (51.2)	5703 (50.1)
	4–5	2788 (23.2)	2453 (23.2)	2655 (23.4)
	≥6	3437 (28.5)	2720 (25.7)	3017 (26.5)
pregnancy wanted	Wanted	1848 (15.4)	9241 (89.7)	7703 (76.0)
	Not wanted	10, 189 (84.6)	1065 (10.3)	2437 (24.0)
Media exposure	Exposed	4348 (36.1)	6060 (50.9)	1355 (11.9)
	Not exposed	7, 689 (63.9)	4528 (49.1)	10,020 (88.1)

Other^a^: catholic and protestant

Across the three DHS surveys, the Proportion of respondents having home delivery was significantly declined from 94.2% in 2005 to 65.7% in 2016. Except for maternal age, region, wealth index, marital status and parity, all other variables listed in the table showed changes in composition, when comparing the sample population in the years 2005, 2011 and 2016 (Table 1).

### Trends in stillbirth rate

**Fig. 1:**
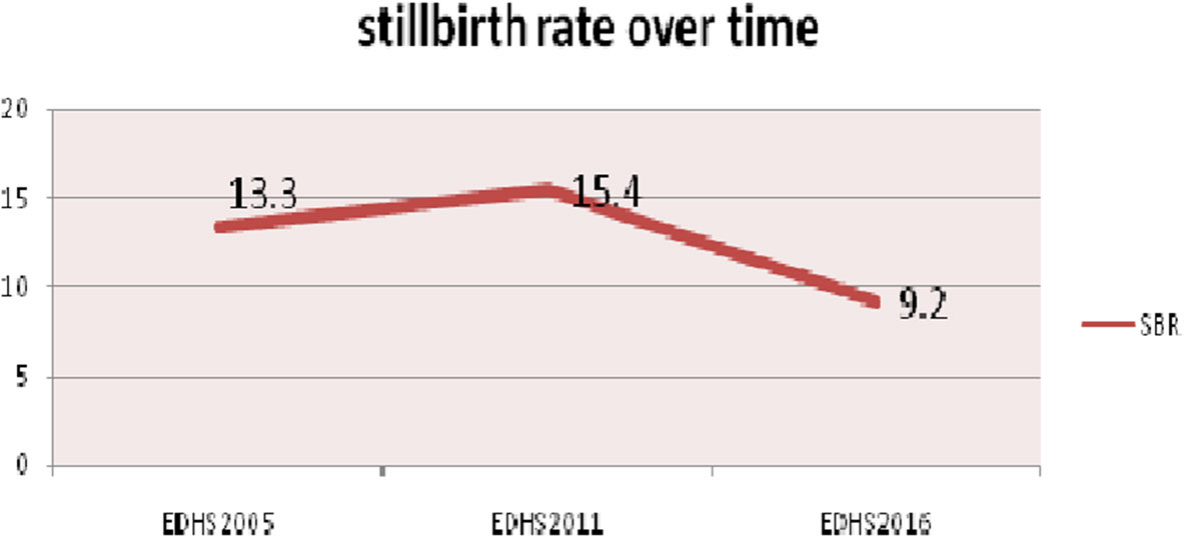
The trends of stillbirth rate in Ethiopia from 2005 to 2016

The trend period was divided into three phases, 2005–2011, 2011–2016 and 2005–2016 to see the differences in stillbirth rate over time and the potential source for the change in SBR. The rate of stillbirth over the study period (2005–2016) has been declined. The largest decline was seen in the second phase (2011–2016) with a 6.2 point change in SBR but in the first phase 2005–2011 there was slight rose from 13.3 [11.4, 15.5] to 15.4 [13.2, 17.9] per 1000 births then decline to 9.2 [7.6,11.1] (Fig. 1). The change in the stillbirth rate over the study period (2005–2016) was significant.

### The trends in stillbirth rate by women’s characteristics

**Fig. 2:**
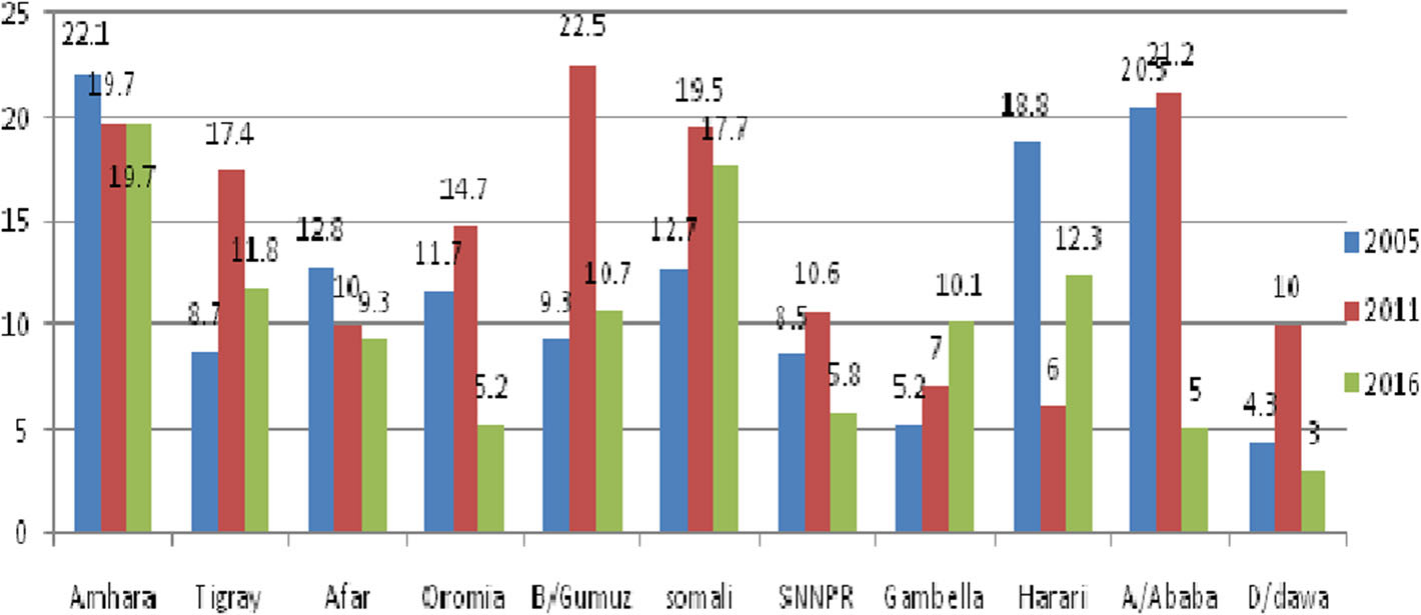
The trends of stillbirth rate over time across regions in Ethiopia 2005, 2011 and 2016

The trend in stillbirth rate by women who gave birth within 5 years before the survey showed variation according to their characteristics (Table 2). Major Decline in stillbirth rate was observed in some of the categories. Based on the region, the stillbirth rate was increased in the first phase at a 13.2 point increase in Benishangul-Gumuz but declined in the second phase at 11.8 point decrease in SBR but stillbirth rate in Amhara region was consistently high over the study period (Fig. 2). Besides, there was a decrease in SBR in women with secondary and higher education with a decline in the third phase of the study period (2005–2016) at a 12.4 point decrease in the Stillbirth rate.

**Table 2:** Trends in stillbirth rate among women’s who gave birth in the last five years prior to the surveys by selected characteristics 2005, 2011, and 2016 Ethiopia Demographic and Health Surveys

Characteristics	2005 *N* = 12037	2011 *N* = 10588	2016 *N* = 11375	point difference in stillbirth rate		
				Phase 1 2011–2005	Phase 2 2016–2011	Phase 3 2016–2005
Residence						
Urban	14.7	6.3	0.8	−8.4	−5.5	−13.9
Rural	13.2	16.8	10.2	3.6	−6.6	−3.0
Religion						
Orthodox	13.4	17.9	12.7	4.5	−5.2	−0.7
Muslim	9.8	14.7	11.0	4.9	− 3.7	1.2
Other*	15.6	12.6	1.3	−3.0	−11.3	−14.3
Maternal age						
< 20	17.4	11.2	17.1	−6.2	5.9	−0.3
20–29	12.9	18.2	9.3	5.3	−8.9	−3.6
30–39	11.5	13.1	7.8	1.6	−5.3	−3.7
40 +	19.4	10.8	11.4	−8.6	0.6	−8.0
Maternal occupation						
Had occupation	9.6	17.5	11.8	7.9	−5.7	2.2
No occupation	14.9	13.7	7.1	−1.2	−6.6	−7.8
Women’s education						
No education	14.9	17.4	10.8	2.5	−6.6	−4.1
Primary education	4.4	11.0	5.9	6.6	−5.1	1.5
Secondary and above	18.8	10.6	6.4	−8.2	−4.2	−12.4
Parity						
1	26.9	32.8	16.3	5.9	−16.5	− 10.6
2–4	13.0	14.1	11.1	1.1	−3.0	−1.9
≥ 5	13.3	12.7	5.1	−0.6	−7.6	−8.2
Birth order						
1–3	16.3	18.0	13.2	1.7	−4.8	−3.1
4–5	6.9	14.8	6.6	7.9	−8.2	−0.3
≥ 6	13.5	10.9	3.8	−2.6	−7.1	−9.7
Place of delivery						
Home	12.7	15.8	8.1	3.1	−7.7	−4.6
Health facility	24.5	9.5	11.3	−15.0	1.8	−13.2
Mode of delivery						
Caesarean section	53.3	23.0	29.9	−33.3	6.9	−23.4
Vaginal delivery	13.0	15.1	8.5	2.1	−6.6	−4.5
Number of ANC visit						
No visit	15.7	21.4	15.4	5.7	− 6.0	−0.3
1–3 visit	13.0	15.9	8.3	2.9	−7.6	−4.7
≥ 4 visit	19.5	11.0	7.0	−8.5	−4.0	−12.5
Maternal anemia						
Yes	8.1	31.9	12.0	23.8	−19.9	3.9
No	14.9	11.9	7.8	−3.0	−4.1	−7.1
Wealth index						
Poorest	10.5	17.7	9.5	6.2	−8.2	−1.0
Poorer	9.7	15.6	11.5	5.9	−4.1	1.8
Middle	13.8	23.5	10.2	9.7	−13.3	−3.6
Richer	19.0	11.6	6.9	−7.4	−4.7	−12.1
Richest	14.1	5.9	6.2	−8.2	0.3	−7.9
BMI						
Thin	12.3	15.0	10.4	2.7	−4.6	−1.9
Normal	13.7	16.3	9.1	2.6	−7.2	−4.6
Overweight	42.3	7.1	6.3	−35.2	−0.8	−36.0
Maternal height						
< 150 cm	13.0	37.3	15.3	24.3	−22.0	2.3
≥ 150 cm	13.0	12.4	8.4	−0.6	−4.0	−4.6
Preceding birth interval						
< 24 month	8.9	10.6	11.8	1.7	1.2	2.9
≥ 24 months	12.1	14.1	8.6	2.0	−5.5	−3.5
Was the pregnancy wanted						
Yes	11.8	15.4	9.6	3.6	−5.8	−2.2
No	13.6	12.6	5.0	−1.0	−7.6	−8.6
Ever use contraceptive						
Yes	20.4	13.5	5.9	−6.9	−7.6	−14.5
No	11.4	17.0	12.0	5.6	−5.0	0.6
Media exposure						
Yes	11.4	15.5	3.3	4.1	−12.2	−8.1
No	16.7	15.4	10.0	−1.3	−5.4	−6.7
Overall	13.3[11.4,15.5]	15.4[13.2,17.9]	9.2[7.6,11.1]	2.1	−6.2	−4.1

*represents protestant, catholic and traditional religion followers

Concerning having 4 and above ANC visits, there was a decline in SBR over the study period with the highest decline during the third phase (2005–2016) with point decline in 12.5 per 1000 birth (Table 2). The rate of stillbirth was declined significantly among women who gave birth at the health facility and among women gave birth through the cesarean section with point decrease of 13.2 and 24.3 per 1000 births respectively over the entire study period. According to the place of residence, the rate of stillbirth was decreased by 13.9 per 1000 births among urban residents from 2005 to 2016 (Table 2).

### Decomposition analysis

#### Decomposition analysis of stillbirth in Ethiopia, 2005–2016

Overall from 2005 to 2016, there has been a significant decline in the stillbirth rate in Ethiopia. The overall decomposition result showed that the decline in stillbirth over time has been explained by the differences in women’s characteristics between the surveys. About 82.3% of the decrease in stillbirth was attributed to the differences in the composition of the respondent but the change due to the differences in the effect of selected explanatory variables was not significant (Table 3).

**Table 3:** Overall decomposition analysis of change in stillbirth in Ethiopia 2005–2016

Stillbirth	Coef.	[95% Conf. Interval]	Pct.
E	−.0053	−0.0098 -0.00081	82.3*
C	−.00113	− 0.008 0.006	17.7
R	− 0.0064	− 0.001-0.0017**	

^*^E:endowment; C:coefficient; R: residual; * *p*-value< 0.05; **p-value< 0.01

In the detailed decomposition analysis, the overall decrease in stillbirth between 2005 and 2016 was attributed to the differences in characteristics (endowment) of women between the surveys. The most important independent variables that provide significant contributions were the mode of delivery, place of delivery, and place of residence. The increase in the composition of women with urban residence from 2005 to 2016 was significantly contributed to the decrease in stillbirth. Also, an increase in the composition of women with health facility delivery over time (from 2005 to 2016) was significantly contributed to the decrease in stillbirth, which contributes 19.3%. Similarly, an increase in the composition of women with cesarean delivery over time (from 2005 to 2016) significantly contributed to the change in stillbirth about 1.4% of the change in stillbirth (Table 4).

**Table 4:** Detailed decomposition analysis of change in stillbirth in Ethiopia 2005–2016

Experiencing stillbirth		Difference due to characteristics(E)		Difference due to coefficient (C)	
		Coef.	Pct.	Coef.	Pct.
Residence	Rural	−0.0001** [− 0.0001,-0.002]	0.9	0.006[− 0.03, 0.04]	−94.6
	Urban				
Maternal education	No education				
	Primary education	−0.0007 [−0.002,0.0001]	10.4	0.0003 [−0.002, 0.002]	−5.1
	Secondary and higher	−0.000003[− 0.00003,0.0002]	0.05	0.00001 [− 0.0003, 0.0004]	−0.9
Mode of delivery	Vaginal delivery				
	Caesarean section	0.00009*[0.00002,00002]	−1.4	0.00004 [−0.0002, 0.0003]	−0.6
Place of delivery	Home				
	Health facility	0.0012* [0.0003,0.002]	−19.3	0.00006 [−0.0004, 0.0005]	−0.9
Parity	Only one birth				
	2–4 birth	−0.00007 [− 0.0003,0.0002]	1.1	0.0005 [− 0.002, 0.003]	−7.0
	≥5 birth	−0.0002 [− 0.0004,00001]	2.8	− 0.000525[− 0.004, 0.002]	8.2
use contraceptive	Yes				
	No	−0.0013 [− 0.002,0.000002]	20.7	0.003 [− 0.012, 0.018]	−45.2
Wealth status	Poor				
	Middle	−0.00005 [− 0.0003,0.00002]	0.1	− 0.00009 [− 0.0009,0.0007]	1.4
	Rich	0.00003[−.00002,0.0002]	−0.4	− 0.00064 [− 0.004, 0003]	9.9
Number of ANC visit	No ANC visit				
	1–3 visit	−0.0003 [− 0.0007,0.0001]	4.5	− 0.0002 [− 0.001, 0.0008]	2.9
	≥4 visit	−0.003 [− 0.006, 0.0002]	41.4	−0.0003 [− 0.002, 0.001]	5.1
total			82.3		17.7
Constant				−0.009[−0.06, 0.04]	

* *P*-value< 0.05; ** *p*-value< 0.01

## Discussion

Stillbirth is a major but often overlooked public health issue [28]. The incidence of stillbirth in a community is a reflection of the level of antenatal care and delivery service availability and utilization [29]. The study investigated the trends and determinants of stillbirth among reproductive-age women in Ethiopia. The study aimed to identify the major factors positively or negatively contributing to the change in stillbirth rate in the past 10 years based on data from 2005, 2011 and 2016 Ethiopian Demographic and Health Surveys.

In this study, the trend in the stillbirth rate has been significantly declined over time. The stillbirth rate declined from 13.3/1000 birth in 2005 to 9.2/1000 births with an annual reduction rate of 3.1%. Which is in line with a systematic review done in Ethiopia [9]. This could be attributed to the establishment of health extension workers that improve maternal and child health service delivery in the district by delivering appropriate information about the different available services to the mothers at the community and household level and health development army to facilitate access to basic maternal health service especially for rural residents [30]. Besides, in Ethiopia Antenatal Care (ANC) utilization is increased through time [31]. This could help a pregnant woman to seek early treatment for her potential pregnancy-associated complications, early screening of underlying medical conditions and may improve birth outcomes by promoting deliveries in health facilities where complications can be better managed and have access to information related to nutrition, and danger signs of pregnancy [32]. Based on this study result Ethiopia achieved the Every Newborn Action plan set by WHO, a global multi-partner movement to reduce stillbirth rates globally to 12 or less per 1000 births by 2030 [11, 33].

In the decomposition analysis, the stillbirth rate (2005–2016) has shown a remarkable decrement in Ethiopia. Hence, understanding the source of change has public health importance to uncover what are the contributing factors for the change in stillbirth as well as understanding where we are making progress in reducing stillbirth to evaluate already implemented strategies and to work on factors contributing to the change in the trend of stillbirth.

82.3% of the decreases in stillbirth rate over the entire study period (2005–2016) was attributed to the differences in women’s composition over the surveys. An increase in the composition of urban resident women over the survey showed a significant effect on the decrease in stillbirth. This finding is consistent within South Africa [34], African Great lake Regions [35], Nigeria [20], Northern Ghana [23] and Ethiopia [24]. This could be due to the increase in urbanization in Ethiopia over time [36] and increased urbanization has significant role in improving access to maternal health service especially emergency obstetric cases can be managed since they are near to the health facilities, and had relatively improved awareness towards maternal health service utilization as compared to rural residents [37].

An increase in cesarean delivery over time had a significant effect on the decline of stillbirth over time. This finding was in line with prior study in England [38], this could be the fact that cesarean delivery is done in advanced setting and the health facilities are increasing over time in Ethiopia. Therefore intrapartum stillbirth can be managed in emergency cases through the cesarean section in situations like cord prolapse, placental Previa and fetal distress and could save the baby [39].

Besides, an increased health facility delivery over the survey’s had a significant effect on the decline in stillbirth. This might be due to the reason that most of the stillbirth are occurred during intrapartum period [40] due to complications arising during labor this could be managed for women who gave birth at health facility since there are skilled birth attendants, emergency care is available at health facility and access to quality obstetric care at the time of delivery can reduce preventive stillbirth this could be the possible reason.

The limitations of the study were, the EDHS data didn’t measure many other important factors, for example, those related to maternal health service availability and quality, maternal medical and obstetric conditions: Variables like Diabetes mellitus, Hypertension, HIV/AIDS, heart failure, renal diseases, and maternal obstetric complications such as Antepartum hemorrhage, preeclampsia, eclampsia, premature rupture of membrane, polyhydramnios, IUGR, preconception BMI, folate supplementation before pregnancy, congenital anomalies, ABO incompatibility, women knowledge and attitude towards stillbirth, RH iso-immunization which are considered as the most common cause of Stillbirth were not addressed in this study because these variables were not available. While decomposition analysis is a promising tool to analyze contributions of various factors to changes in outcome, our model is constrained by the limited availability of data to explain the difference. Further research is needed including an alternative methodology to the decomposition analysis.

## Conclusions

Stillbirth rates had shown a remarkable decrease over the last 10 years in Ethiopia. More than 3/4th of the overall decrease in stillbirth rate among reproductive-age women over the 10 years was due to differences in characteristics of respondents between 2005 and 2016 EDHS. Changes in the composition of reproductive age women’s characteristics according to the place of residence, mode of delivery and place of residence were the major sources of the decrease in stillbirth rate over the study. Public health interventions, including strengthening health facility delivery for women to further reduce stillbirth particularly for rural residents.

## Data Availability

Data is available online and you can access it from www.measuredhs.com

## Abbreviations

ANC: Antenatal Care
ARR: Annual Rate of Reduction
BMI: Body Mass Index
CI: Confidence Interval
CSA: Central Statistical Agency
DHS: Demographic Health Survey
EA: Enumeration Area
EDHS: Ethiopian Demographic Health Survey
PHC: Population and Housing census
SBR: Stillbirth Rate
SNNPRs: Southern Nations and Nationality People Regional state
WHO: World Health Organization
